# Primary Products from Fast Co-Pyrolysis of Palm Kernel Shell and Sawdust

**DOI:** 10.3390/molecules28196809

**Published:** 2023-09-26

**Authors:** David O. Usino, Päivi Ylitervo, Tobias Richards

**Affiliations:** Swedish Centre for Resource Recovery, University of Borås, 501 90 Borås, Sweden; paivi.ylitervo@hb.se (P.Y.); tobias.richards@hb.se (T.R.)

**Keywords:** fast pyrolysis, primary products, co-pyrolysis, Py-GC-MS/FID, biomass blend

## Abstract

Co-pyrolysis is one possible method to handle different biomass leftovers. The success of the implementation depends on several factors, of which the quality of the produced bio-oil is of the highest importance, together with the throughput and constraints of the feedstock. In this study, the fast co-pyrolysis of palm kernel shell (PKS) and woody biomass was conducted in a micro-pyrolyser connected to a Gas Chromatograph–Mass Spectrometer/Flame Ionisation Detector (GC–MS/FID) at 600 °C and 5 s. Different blend ratios were studied to reveal interactions on the primary products formed from the co-pyrolysis, specifically PKS and two woody biomasses. A comparison of the experimental and predicted yields showed that the co-pyrolysis of the binary blends in equal proportions, PKS with mahogany (MAH) or iroko (IRO) sawdust, resulted in a decrease in the relative yield of the phenols by 19%, while HAA was promoted by 43% for the PKS:IRO-1:1 pyrolysis blend, and the saccharides were strongly inhibited for the PKS:MAH-1:1 pyrolysis blend. However, no difference was observed in the yields for the different groups of compounds when the two woody biomasses (MAH:IRO-1:1) were co-pyrolysed. In contrast to the binary blend, the pyrolysis of the ternary blends showed that the yield of the saccharides was promoted to a large extent, while the acids were inhibited for the PKS:MAH:IRO-1:1:1 pyrolysis blend. However, the relative yield of the saccharides was inhibited to a large extent for the PKS:MAH:IRO-1:2:2 pyrolysis blend, while no major difference was observed in the yields across the different groups of compounds when PKS and the woody biomass were blended in equal amounts and pyrolysed (PKS:MAH:IRO-2:1:1). This study showed evidence of a synergistic interaction when co-pyrolysing different biomasses. It also shows that it is possible to enhance the production of a valuable group of compounds with the right biomass composition and blend ratio.

## 1. Introduction

Fast pyrolysis can be used to convert biomass into bio-oil and chemicals. The pyrolysis oil (often denoted as bio-oil when the material is of biomass origin) is composed of compounds such as anhydrosugars, furans, alcohols, ketones, aldehydes, acids, and phenols [1,2]. However, the bio-oil cannot be used directly in fuel engines or mixed with petroleum products due to its high water content, high acidity, and low miscibility, and, therefore, it requires a further upgrade to make it suitable as transportation fuel [1,3]. Several attempts have been employed to improve the quality of the oil. These include the use of catalysts and hydrogen [4,5]. However, these processes are complex and costly due to the equipment requirement and the catalysts needed for the successful upgrade [4,5]. The pretreatment of biomass with dilute acid solutions has also been used to improve the quality and minimise the negative effects of inorganic materials during the fast pyrolysis of biomass [6,7,8]. Recently, attention has been directed towards the co-pyrolysis of different biomasses [9,10,11,12].

The co-pyrolysis of biomasses is described as the pyrolysis of blends, including two or more different biomasses. Previous studies have shown this to improve the overall quality of the pyrolysis oil, such as by increasing the calorific value and promoting the yield of volatile compounds [4,12,13,14,15]. Many studies have been carried out on the co-pyrolysis of different biomasses to produce biofuel and chemicals. However, most of the studies found in the literature focused on the co-pyrolysis of biomass and plastic [16,17,18,19], and very few studies have been carried out on the co-pyrolysis of different biomass materials [10,20,21,22,23]. Moreover, most of the studies that were carried out on the co-pyrolysis of different biomasses were achieved with the use of the TGA and focused on the gas and char yields. For example, El-Sayed et al. [22] showed that the co-pyrolysis of Egyptian olive pomace and wood dust (Kroneiki olive-pomace (KROP), Shamlali olive-pomace (SHOP), and Fine Swedish sawdust (FSSD)) showed an increase in the amount of volatile matter in the blend and had the best synergistic pyrolysis performance at a heating rate of 10 °C/min. Additionally, Nie et al. [10] observed that the co-pyrolysis of wood sawdust (WS) and peanut shell (PS) resulted in an increase in the comprehensive pyrolysis index for the blend ratio W3P7 (WS:PS = 3:7) compared to the single pyrolysis of WS and PS at a heating rate of 10–30 °C/min, while Ge et al. [23] did not observe any clear synergic interaction on the biomass mass loss during the co-pyrolysis of pine wood waste and straw waste. In terms of the bio-oil yield, Biswas et al. [20] showed that the co-pyrolysis of Phumdi (PH) and Para grass (PG) (1:1) resulted in a bio-oil yield of 11.66 wt% and was composed mainly of phenolic compounds, while Hopa et al. [14] observed that the co-pyrolysis of rice husk and sugarcane bagasse resulted in an increased yield of bio-oil with 28.4%. They suggested that this was due to a synergistic interaction between the two biomasses. However, several factors can influence the yield and quality of the pyrolysis product formed from mixing two or more biomasses. These include the biomass type, composition, blending ratio, reactor type, and temperature [4,24]. Tauseef et al. [25] observed a synergistic effect when coal and rice husk were co-pyrolysed. Edmunds et al. [3], in contrast, found that the pyrolysis product distribution was a simple linear combination when switch grass and pine residue were co-pyrolysed. The mixing as well as the blend ratios are important to estimate the final resulting composition and yield of the bio-oil. Furthermore, biomasses differ in their compositions and physical structures, and this can influence the quality of the bio-oil [3]. Palm kernel shell (PKS), for example, has a high lignin content (≈58 wt%) [8,24] compared to woody biomass (15–40%) [26], while woody biomass, such as mahogany (MAH), has a higher carbohydrate content (65 wt%) [27]. During the pyrolysis of raw biomass, cellulose and hemicellulose undergo dehydration, depolymerisation, and rearrangement reactions to form anhydrous sugars, furans, and light oxygenate compounds [28], while lignin undergoes depolymerisation, demethylation, and fragmentation reactions to form mainly phenolic-type compounds [29]. The co-pyrolysis of these biomasses could result in a synergistic effect that could either enhance or decrease the primary products formed. A comparison of the co-pyrolysis of a blend from pure cellulose, hemicellulose, and lignin with the pyrolysis of native birch wood shows a decrease in the product yields of the sugars and phenolic compounds from the native birch wood, while the yields of the hemicellulose-derived products, such as aldehydes and ketones, were promoted [30]. The decreased yield of the sugars, especially levoglucosan, is suggested to depend on the presence of a covalent bond in the morphology of the native biomass and inorganic materials in the native biomass [30].

A review of most of the previous studies shows that the information is scarce about the primary products’ characteristics and interactions during the fast co-pyrolysis of different native biomass blends comprising an agricultural residue and a woody biomass residue. Moreover, most of the studies were carried out with the use of a fixed-bed and/or thermogravimetric analyser (TGA) [10,20,22,23], which may result in secondary reactions. With the right mixture of biomasses, it may be possible to control the product distribution and enhance the quality of the volatile compounds formed from the interaction of the different biomass components during pyrolysis. This study aims to investigate the interactions between an agricultural residue and two woody biomass materials and their impact on the primary-product distribution. Additionally, this study investigates the influence of the blending ratio. Overall, this study provides a method for reducing the environmental impact associated with the disposal of these wastes and promoting the co-utilisation of these waste streams for bioenergy production.

## 2. Results and Discussion

### 2.1. Characterisation of Biomass

A summary of the proximate analysis in terms of the moisture content, volatile matter, fixed carbon, and ash content, as well as the biomass characterisations for all three biomasses used as blends in this study, are shown in Table 1. The proximate analysis shown in Table 1 provides information about the thermal behaviour and fuel characteristics of the PKS and the two woody biomasses (MAH and iroko (IRO)). It also shows the major components and chemical compositions of the PKS, MAH, and IRO, which are important for characterising the energy content, conversion efficiency, and suitability of each biomass for bioenergy production [14,31]. It can be seen from the proximate analysis that the woody biomass samples had higher volatile matter contents compared to the PKS. Volatile matter is important for understanding the energy content of the biomass. Table 1 shows that the PKS had the highest fixed carbon content and heating value, as well as the lowest moisture and ash content, compared to the woody biomasses. These characteristics make PKS a suitable material for blending and co-pyrolysis. The fixed carbon gives an indication of the energy potential of the biomass, while the high moisture content of a biomass material can negatively influence the energy content and efficiency [32]. The ash content is used to determine the inorganic materials in a biomass fuel, which could act as a catalyst during biomass pyrolysis [32,33]. High ash content has been noted to decrease the yield of bio-oil while, at the same time, increase the char and gas yield [34]. Table 1 also shows that the woody biomass had a higher amount of holocellulose (cellulose and hemicellulose) compared to the PKS, which had a higher content of lignin.

### 2.2. Effect of Blending Two Biomasses on Product Distribution and Yield

**Figure 1:**
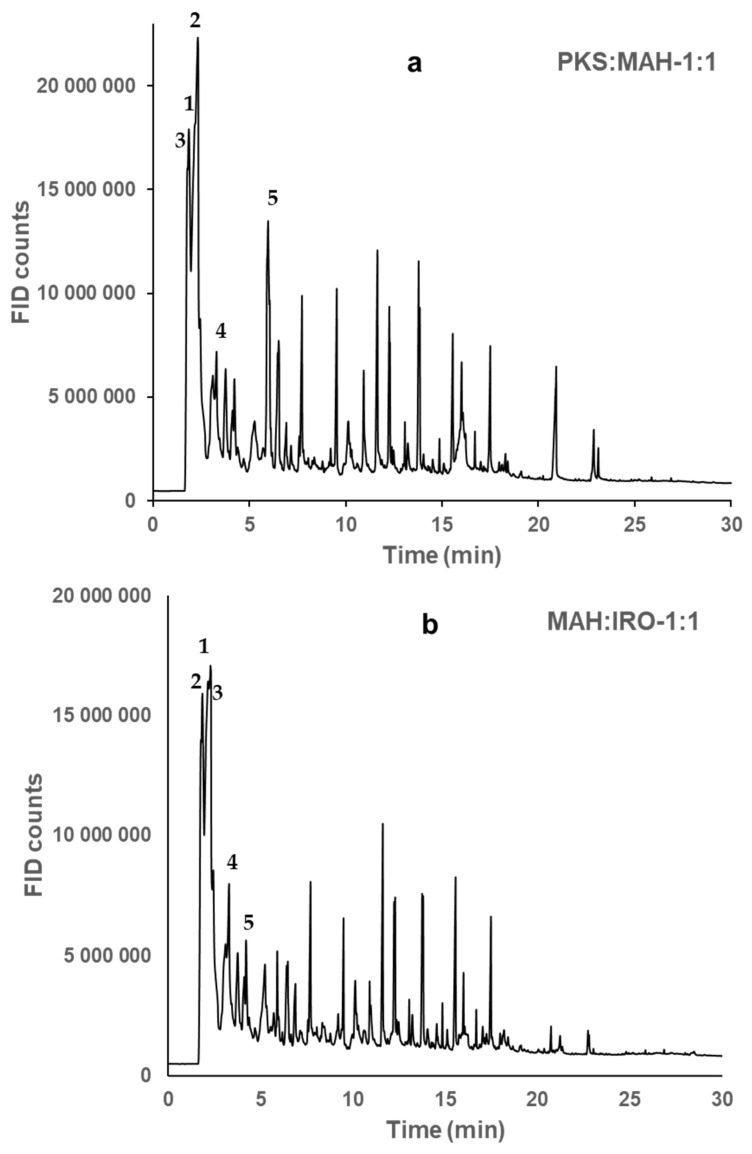
Chromatogram (GC-FID) after co-pyrolysis of two biomass blends at 600 °C and 5 s. The five main components of PKS:MAH-1:1 (**a**) are as follows: (1) hydroxyacetaldehyde, (2) acetic acid, (3) acetaldehyde, (4) 1-hydroxy-2-propanone, and (5) phenol, while those of MAH:IRO-1:1 (**b**) are as follows: (1) hydroxyacetaldehyde, (2) acetaldehyde, (3) acetic acid, (4) 1-hydroxy-2-propanone, and (5) 2,3-pentanedione. NB: (**a**) represents GC-FID chromatogram for PKS:MAH-1:1 and PKS:IRO-1:1, as both blends showed similar primary-product distributions.

The product distribution from the pyrolysis of the individual biomasses and their blends are found in Table S1 in the supplementary material. The pyrolysis of the two biomass blends was achieved by blending them in equal proportions: PKS:MAH_1:1 (0.50:0.50), PKS:IRO_1:1 (0.50:0.50), and MAH:IRO_1:1 (0.50:0.50). It should be noted that the hydroxyacetaldehyde (HAA) and acetic acid (AA) peaks were observed to co-elute for the blended biomasses, as shown in Figure 1. These two peaks (HAA and AA) were observed as the most pronounced peaks in the chromatogram and were resolved by magnifying the chromatographic peak area using the same baseline in order to determine the fraction of the peak area for each chemical compound formed. Hence, the predominant volatile compounds formed when PKS is co-pyrolysed with MAH or IRO (Figure 1a) are as follows: (1) HAA, (2) AA, (3) acetaldehyde, (4) 1-hydroxy-2-propanone, and (5) phenol. Additionally, the main chemical compounds identified when the two woody biomasses (MAH:IRO-1:1) are co-pyrolysed (Figure 1b) are as follows: (1) HAA, (2) acetaldehyde, (3) AA, (4) 1-hydroxy-2-propanone, and (5) 2,3-pentanedione. It can be observed in Table S1 that the relative yield of AA and phenol was two times larger when PKS was co-pyrolysed with either MAH or IRO compared to when the two woody biomasses were co-pyrolysed. This may be attributed to the high lignin content of the PKS, as shown in Table 1, which was the main source of the phenol formation and its interaction with the holocellulose composition of the woody biomass. Previous studies suggest that the amount of AA formed during pyrolysis is dependent on the degree of the acetylation of the biomass feedstock, especially biomasses with high lignin contents [35,36]. Another study showed that lignin contributed to the increased production of AA during the wet oxidation of lignocellulosic biomass [37]. This clearly shows that the types of biomasses used in co-pyrolysis influence the yield and the chemical compounds formed.

**Figure 2:**
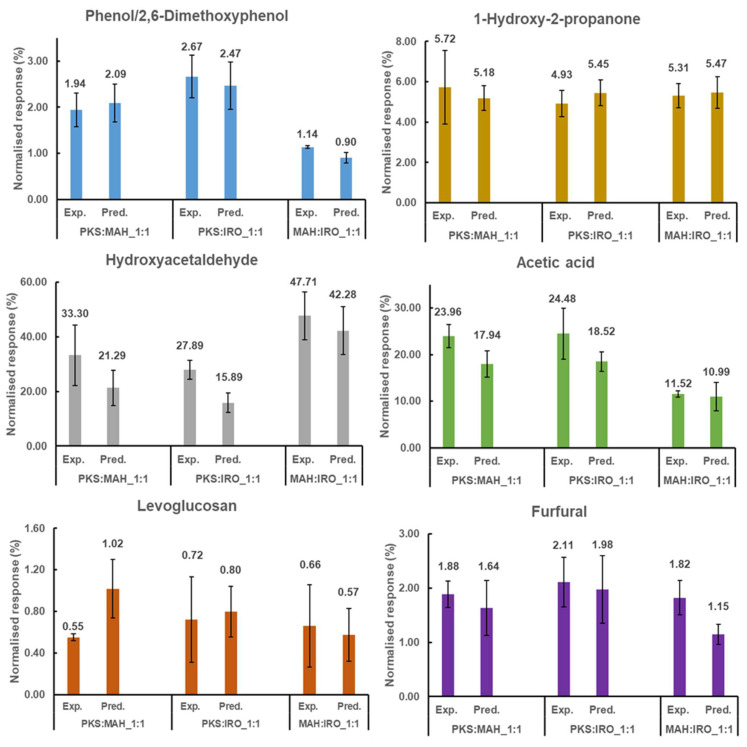
Comparison of the experimental (Exp.) and predicted (Pred.) results of the six main chemical compounds formed for the different classes of compounds after the co-pyrolysis of two different biomass blends. Product distribution is given in normalised weight (%) based on the calculated response (count/µg sample). Phenol is the main compound formed among the phenols during the pyrolysis of PKS:MAH-1:1 and PKS:IRO-1:1, while 2,6-Dimethoxyphenol is the main compound for MAH:IRO-1:1.

To investigate the synergistic effects on the product yield of the co-pyrolysed biomass blends, the yield based on pure biomass according to the blend ratio was added (the so-called predicted value) and compared to the measured value (the experimental value). An analysis of the volatile compounds formed for the two biomass blends during pyrolysis, as shown in Figure 2 and Table S1, indicates that the six main chemical compounds formed when PKS is co-pyrolysed with MAH or IRO for each class of biomass are as follows: phenol (phenols), 1-hydroxy-2-propanone (ketones), hydroxyacetaldehyde (aldehydes), acetic acid (acids), levoglucosan (saccharides), and furfural (furans). However, in contrast to the formation of phenol as the main phenolic compound formed during the co-pyrolysis of PKS with MAH or IRO, 2,6-dimethoxyphenol was observed as the main phenolic compound formed for the pyrolysis of the woody biomass (MAH:IRO-1:1). This can be attributed to the composition and structural differences in the lignin between the PKS and the two woody biomasses. The pyrolysis of PKS produced a higher yield of guaiacyl compounds, while the pyrolysis of either MAH or IRO produced a higher yield of syringyl compounds (see Table S1). For the two component blends, the relative yield of AA was slightly promoted for PKS:MAH-1:1, while HAA was promoted by 43% for PKS:IRO-1:1. AA is formed mainly by the deacetylation of hemicellulose [1,24], or by the thermal cracking of cellulose and depolymerisation of the lignin polymer, while HAA is mainly formed by the primary decomposition and ring cleavage of the glucosidic bond of the cellulose and hemicellulose monomers in the biomass [28]. The increment in the relative yield of the AA may be due to the acetylation of lignin during the pyrolysis of PKS and MAH and the presence of alkali and alkaline earth metals (AAEMs). The presence of inorganic materials, such as AAEMs, in the biomass blend could act as a catalyst that may enhance the production of organic acids, such as AA and other light oxygenated compounds [3]. Pan et al. and Richards et al. [38,39] showed that the yields of AA and HAA were promoted mainly by the presence of potassium and calcium during the pyrolysis of cottonwood. This implies that the AAEMs inherent in the biomass have a strong catalytic effect on the production of AA. Figure 2 also shows that the relative yields of 2,6-dimethoxyphenol and furfural were promoted by 21 and 37%, respectively, for the pyrolysis of the MAH:IRO-1:1 blend, while no difference was observed in the relative yields for 1-hydroxy-2-propanone, HAA, and AA. This may be due to the structural similarity and higher content of holocellulose in the woody biomasses for MAH and IRO, as shown in Table 1, when compared to PKS.

The relative yields of phenol, 1-hydroxy-2-propanone, and furfural for PKS co-pyrolysed with either MAH or IRO in equal proportions showed no difference. However, the relative content of levoglucosan was strongly inhibited for the pyrolysis of PKS:MAH-1:1. The inhibition of levoglucosan for the PKS:MAH-1:1 blend and the promotion of AA, and HAA when PKS is co-pyrolysed with MAH or IRO, as well as the enhancement of 2,6-dimethoxyphenol and furfural for the co-pyrolysis of the woody biomass, are evidence of a synergistic interaction of the biomass components.

**Figure 3:**
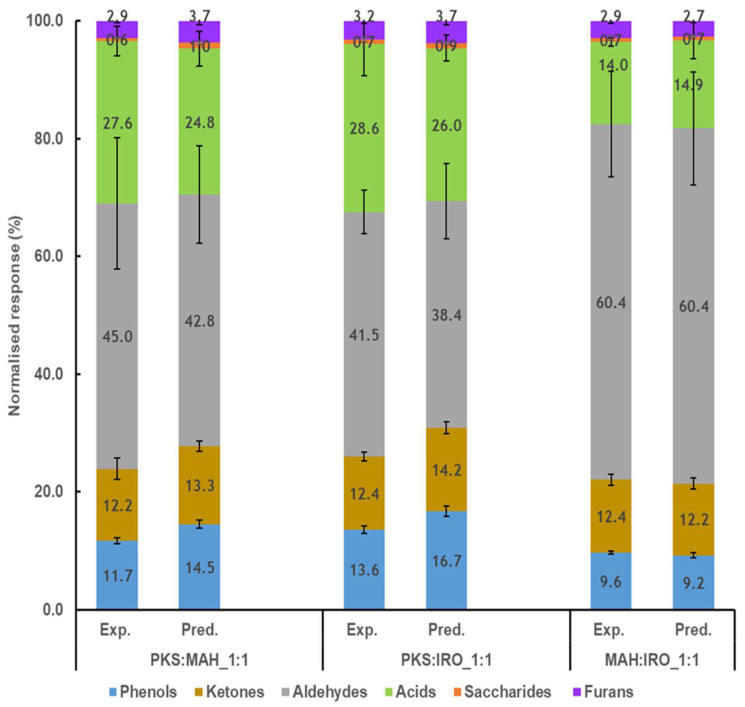
Comparison of the experimental (Exp.) and predicted (Pred.) results of the different classes of compounds after co-pyrolysis of two biomasses.

The experimental (Exp.) and predicted values (Pred.) for the different classes of compounds are presented in Figure 3. It was found that the relative content of the phenols was inhibited by 19% when PKS was co-pyrolysed with either MAH or IRO in equal proportions (PKS:MAH-1:1 and PKS:IRO-1:1). The suppression of the phenols observed may be due to the interaction between the different biomass components and the presence of inherent metal oxides in the biomass. Zhang et al. [40] observed that the yield of the phenolic compounds formed during the pyrolysis of pure cellulose and lignin impregnated with K_2_O and CaO decreased, and the decreased yield was most pronounced for the guaiacol and 4-allyl-2-6-dimethoxyphenol compounds. Chang et al. [41] also observed a decrease in the relative content of the phenols when PKS and *Nannochloropsis* sp. (NC) were co-pyrolysed at 600 °C with a blend ratio of 1:1. The inhibition of the phenols is important for improving the quality of the bio-oil, as they contribute to the instability of the bio-oil during storage and transport [42]. No difference was found in the relative yields for the furans, acids, and aldehydes for the co-pyrolysis of PKS with either MAH or IRO. The relative yield of the saccharides, although very low in relation to the total amount, was strongly inhibited for the pyrolysis of PKS:MAH-1:1. The inhibition of the saccharides, especially levoglucosan, observed for the pyrolysis of PKS:MAH-1:1 may also be attributed to the presence of inorganic materials, such as K and Ca, in both the PKS and the woody biomass, and the interaction of holocellulose with lignin [39]. The major metal ions found in PKS are Si, K, and Ca, while those of the woody biomass (iroko) are K and Ca [43,44]. Richards et al. [39] observed that the yield of levoglucosan was negatively affected during the pyrolysis of cotton wood, and they attributed this to the presence of metal ions, such as K, Li, and Ca, and the interaction of levoglucosan formation with lignin [39]. Their result is similar to that presented by Usino et al. [30], who investigated the co-pyrolysis of pure cellulose, hemicellulose, and lignin to mimic a native birch wood. They observed that the yields of saccharides and phenols were inhibited to a large extent; moreover, they attributed this to the presence of inorganic materials and the formation of active compounds from the hemicellulose unit, which may have reacted with compounds from the cellulose and lignin and subsequently inhibited their reaction. The inhibition of the phenols and sugars observed in this study may be attributed to the inherent inorganics present in these biomasses and the higher content of lignin present in the PKS that may have reacted with the holocellulose in the woody biomass during pyrolysis. The metal ions present in the biomass are known to induce the hemolytic cleavage of the pyranose ring during the decomposition of cellulose. They are also known to decrease the stability of the glycosidic bonds and the hydroxyl group during pyrolysis, thereby resulting in dehydration, ring scission, and cracking reactions that favour the formation of low-molecular-weight compounds and the inhibition of levoglucosan formation [15].

Additionally, a comparison of the experimental and predicted values for the woody biomasses (MAH and IRO), co-pyrolysed in equal proportions, indicates that no difference was observed in the relative yield across the different classes of compounds. This could be due to the woody biomasses being composed of similar polysaccharide structures. The result is similar to that presented by Edmunds et al. [3], who observed that the pyrolysis products obtained from the co-pyrolysis of switch grass and pine residue were a simple linear combination of the two biomasses investigated. Generally, Figure 2 shows that whenever PKS is co-pyrolysed with either MAH or IRO, a decrease in the relative contents, especially of the phenols and the saccharides, was observed. This indicates the occurrence of a synergistic interaction between these biomass components.

Considering the total yields of the volatile compounds formed (count/µg sample) from the pyrolysis of the biomass blends PKS:MAH-1:1, PKS:IRO-1:1, and MAH:IRO-1:1, it can be seen that the experimental values were about two times higher than the predicted values (Table S2). The yield (count/µg sample) increased from 4.48 × 10^4^ (predicted value) to 7.53 × 10^4^ (experimental value) for PKS:MAH-1:1, from 3.57 × 10^4^ (predicted value) to 7.36 × 10^4^ (experimental value) for PKS:IRO 1:1, and, finally, from 3.60 × 10^4^ (predicted value) to 6.59 × 10^4^ (experimental value) for MAH:IRO 1:1. This shows that co-pyrolysis could lead to an increase in the amount of volatile compounds formed.

### 2.3. Effect of Blending Three Biomasses on Product Distribution and Yield

The pyrolysis of the three biomass blends was achieved by first blending them in equal proportions (PKS:MAH:IRO-1:1:1 (0.33:0.33:0.33)), and then, secondly, mixing one part of the PKS with two parts each of the woody biomass (PKS:MAH:IRO-1:2:2 (0.20:0.40:0.40)), and thirdly, using two parts of the PKS and one part each of the woody biomass (PKS:MAH:IRO-2:1:1 (0.50:0.25:0.25)).

**Figure 4:**
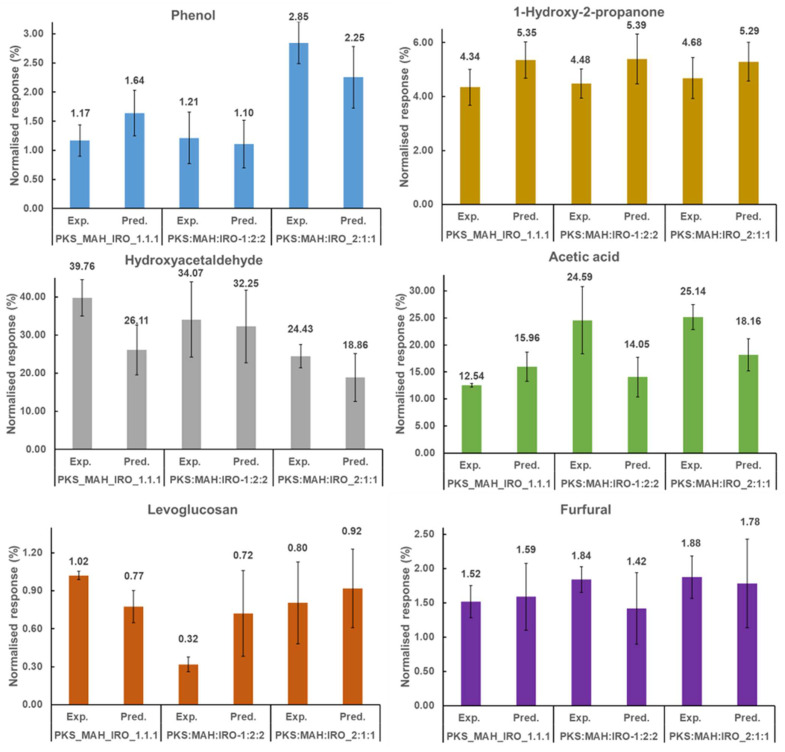
Comparison of the experimental (Exp.) and predicted (Pred.) results of the six main chemical compounds formed after the co-pyrolysis of three different biomass blends. Product distribution is given in normalised weight (%), based on the calculated response (count/µg sample).

Similar to the two biomass blends, the main chemical compounds formed for each class of the three-biomass blend were as follows: phenol (phenols), 1-hydroxy-2-propanone (ketones), HAA (aldehyde), AA (acids), levoglucosan (saccharides), and furfural (furans) (see Figure 4 and Table S1). A comparison of the experimental and predicted values for the main chemical compounds indicates that the relative contents of HAA and levoglucosan were promoted by 34 and 24%, while AA was slightly inhibited for the PKS:MAH:IRO_1:1:1 pyrolysis blend. However, the relative content of AA was promoted for the PKS:MAH:IRO-1:2:2 and PKS:MAH:IRO-2:1:1 pyrolysis blends. It can be observed in Figure 4 that the relative yield of levoglucosan was inhibited when the proportion of the woody biomass was increased in the biomass blend for the co-pyrolysed feedstock (PKS:MAH:IRO-1:2:2). No difference was observed in the relative yield for 1-hydroxy-2-propanone and furfural for any of the three biomass blends, nor for phenol for the PKS:MAH:IRO_1:1:1 and PKS:MAH:IRO-2:1:1 blends. These results show that the composition and blending ratio of the biomasses influence the primary-product distribution when different types of native biomasses are co-pyrolysed.

**Figure 5:**
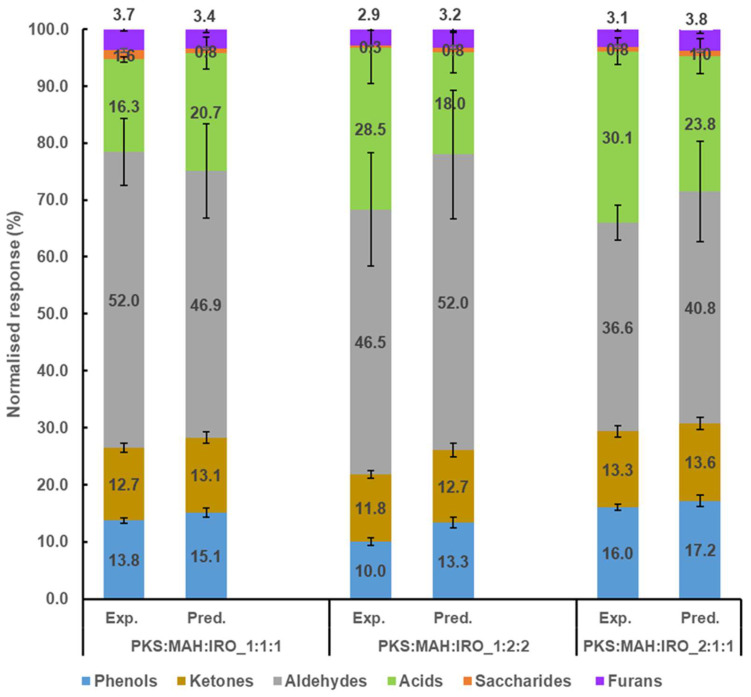
Comparison of the experimental (Exp.) and predicted (Pred.) volatile compositions after co-pyrolysis of three different biomass blends.

Figure 5 shows the effect of blending on the various groups of compounds obtained from the co-pyrolysis of PKS, MAH, and IRO. It was found that the relative content of the saccharides was promoted to a large extent when the three biomasses were blended in equal proportions (PKS:MAH:IRO_1:1:1), which can be attributed to the increased yield of levoglucosan. The promotion may be due to the interaction of free radicals from the PKS and the woody biomass during their co-pyrolysis and the deoxygenation of the PKS-derived oxygenated compounds via the depolymerisation reaction [42]. It should be noted that the total relative yield of the saccharides was low (i.e., less than 2%) in comparison to the total yield of the volatile compounds. However, Table S1 shows that the amount of saccharides (especially levoglucosan) was almost as high for the PKS as it was for the MAH, and it was twice the amount of the IRO. An increased yield of sugars was also observed by Ojha et al. [45], who investigated the fast co-pyrolysis of cellulose and polypropylene at different mass ratios and temperatures. They observed that the yield of anhydrosugars increased with the temperature when cellulose and polypropylene were blended in equal proportions (50:50). However, the relative yield of the saccharides was inhibited to a large extent for the PKS:MAH:IRO-1:2:2 pyrolysis blend. This decrease can be attributed mainly to the inhibition of levoglucosan formation (see Figure 4). Additionally, the relative yield of the acids was promoted for the PKS:MAH:IRO-1:2:2 and PKS:MAH:IRO-2:1:1 pyrolysis blends, while that of the PKS:MAH:IRO-1:1:1 pyrolysis blend was slightly inhibited. The inhibition of the acids observed for the PKS:MAH:IRO-1:1:1 pyrolysis blend may be due to the deoxygenation of the acid via the decarboxylation reaction [42]. It has previously been reported that the strong presence of carboxylic acids in the bio-oil, such as AA, could lead to corrosion in the pipes and burners [46,47]. Moreover, the relative yield of phenols was inhibited by 25% for the PKS:MAH:IRO-1:2:2 pyrolysis blend, while only a slight inhibition was observed for the PKS:MAH:IRO_1:1:1 pyrolysis blend. However, no difference in the yield was observed for the ketones, aldehydes, and furans for any of the three biomass pyrolysis blends (PKS:MAH:IRO_1:1:1, PKS:MAH:IRO-1:2:2, and PKS:MAH:IRO-2:1:1). The increased and decreased yields for the different biomass pyrolysis blends are indications of a synergistic interaction between the different biomass components and could be due to the presence of inorganic materials in the biomasses, which may have acted as a catalyst during the pyrolysis of the blended biomasses [30]. The inhibition of the phenols observed in this study is similar to that reported in a previous study [30], in which it was observed that the relative yield of the phenolic compounds was inhibited when the individual biomass components of cellulose and hemicellulose were co-pyrolysed with lignin. The inhibition of the phenolic compounds was attributed to the presence of inorganic materials and the formation of active sites from the hemicellulose unit inhibiting the decomposition of the cellulose and lignin pyrolysis [30]. Vasu et al. [24] also reported that the amount of lignin-derived compounds was inhibited when the proportion of PKS was reduced in the co-pyrolysis of PKS and palm oil sludge. Nonetheless, the palm oil sludge was reported to have low volatile matter (48 wt.%) and high ash content (24 wt.%) compared to the high volatile matter (≈81%) and low ash content (≈3.7%) of the woody biomasses used in the current study. The blending and co-pyrolysis of the PKS, with a very low ash content (0.9 wt.%), and the woody biomass, with a higher ash content (2.6–4.8%), implies that there was a higher proportion of inorganic materials in the co-pyrolysed biomasses. This increase may have induced catalytic reactions and negatively affected the total yield of the phenolic compounds. The differences in the results among the different blends may be due to interactions as a result of the chemical reactions of the different components and physical action, such as the blend ratio [15]. The results show that the blending ratio plays a vital role in the product yield, and the PKS:MAH:IRO-1:1:1 blend produced volatile compounds with better fuel qualities than the PKS:MAH:IRO-1:2:2 and PKS:MAH:IRO-2:1:1 blends.

The results from the three biomass blends showed an impact on the primary pyrolysis product. While the yield of the saccharides was promoted when the three biomasses were blended in equal promotion and co-pyrolysed, an increase in the woody biomass composition resulted in a decreased yield of levoglucosan. Additionally, the synergistic interaction was more pronounced when the blend ratio of PKS was lower in the blend, as seen in the PKS:MAH:IRO_1:1:1 and PKS:MAH:IRO-1:2:2 (i.e., PKS:woody biomass_1:2 and 1:4) blends, than when they were blended and co-pyrolysed in equal proportions, as seen in the PKS:MAH:IRO-2:1:1 (i.e., PKS:woody biomass_2:2) for the ternary blend. An analysis of the volatile compounds formed in response (count/µg sample), shown in Table S3, shows that the total yield of the volatile compounds formed for the PKS:MAH:IRO_1:1:1 blend was similar for both the experimental and predicted values compared to the PKS:MAH:IRO-1:2:2 and PKS:MAH:IRO-2:1:1 blends, in which the total yield of the volatile compounds formed (experimental value) was promoted by almost two times the predicted value. This may have been the reason for the improved quality of the products from the PKS:MAH:IRO_1:1:1 blend in comparison to the other blends. This study thus shows that there is an interaction during the co-pyrolysis of different biomasses, and the interaction is dependent on the biomass type/composition and blend ratio. This makes it possible to optimise the pyrolysis process with the selective production of specific valuable chemicals.

## 3. Materials and Methods

### 3.1. Materials and Sample Preparation

The materials used for this study were palm kernel shell (*Elaeis guineensis*), mahogany (*Khaya ivorensis*) sawdust, and iroko (*Chlorophora excelsa*) sawdust. These biomasses were selected because of the environmental concerns associated with their disposal, the volume of the produced waste, and their suitability as feedstock for thermochemical conversion. The woody biomasses (MAH and iroko (IRO) sawdust) were collected from sawmills, while the PKS was obtained from a palm oil site, all in the southern parts of Nigeria. The three biomass samples were milled in a cutting mill (Retsch SM 100) with a screen size of 6 mm and sieved to a particle size of 125–250 µm by a Haver (EML 200 pure) test sieve shaker. The samples were then dried in an oven at 105 °C overnight to a constant weight and stored for further use. The biomasses were then blended into binary and tertiary blends. The binary blends were made from equal amounts of two samples, while the tertiary blends were made from all three samples, but in different proportions. The binary blends correspond to the following weight proportions: PKS:MAH_1:1 (0.50:0.50), PKS:IRO_1:1 (0.50:0.50), and MAH:IRO_1:1 (0.50:0.50), while the tertiary blends correspond to the following weight ratios: PKS:MAH:IRO_1:1:1 (0.33:0.33:0.33), PKS:MAH:IRO_1:2:2 (0.20:0.40:0.40), and PKS:MAH:IRO_2:1:1 (0.50:0.25:0.25).

### 3.2. Proximate Analysis

The proximate analysis included the moisture content and the volatile matter of the biomass, and these were determined in reference to the ASTM standard test methods: ASTM E 871-82 and ASTM E872-82 [48,49]. For the moisture content, 3.0 g of each biomass sample was dried in an oven at 105 °C for 16 h, and the weight difference between the raw and dried biomass samples was used to calculate the weight loss of the biomass. Additionally, 1.0 g of each biomass sample was heated in a furnace at a constant temperature of 950 ± 20 °C for 7 min and cooled in a desiccator to determine the volatile matter of the raw biomass. The Nabertherm B 150 furnace was used to determine the ash content of the raw biomass by heating it at 550 °C for 4 h, according to the E1755-01 standard [50]. Prior to heating the biomass, 4.0 g of raw biomass was dried in an oven overnight at 105 °C. The residue left after the combustion of the raw biomass in the furnace was used to calculate the ash content of the biomass. Finally, the fixed carbon was calculated by subtracting the moisture content, volatile matter, and ash content from 100%.

### 3.3. Compositional Analysis of Biomass

Carbohydrate and lignin contents of PKS, MAH, and IRO were determined according to the NREL protocol (NREL/TP-510-42618) [51]. In this procedure, 0.3 g of the biomass sample was mixed with 3 mL 72% sulfuric acid solution. The mixture was stirred every 10 min for 1 h in a water bath at a temperature of 35 °C. Thereafter, 84 mL of Milli-Q water was added to the mixture before it was placed in an autoclave at 121 °C for 60 min. Filtration of the sample was carried out after cooling at room temperature. The collected filtrate was stored in a 50 mL bottle before analysing the acid-soluble lignin and cellulose contents. A spectrophotometer was used to determine the acid-soluble lignin. This was determined by drying the residue overnight at 100 °C and weighing the lignin content after cooling. High-performance liquid chromatography (HPLC) was used to determine the glucose content, while the hemicellulose content was determined by calculating the difference. The column (Aminex HPX-87P, Bio-Rad, Hercules, CA, USA) used was set at 85 °C, and the eluent flow was 0.6 mL/min. The experiments were carried out in duplicates, and the values are reported as average values.

### 3.4. Calorific Analysis

The calorific values of the biomass samples were determined with a bomb calorimeter (IKA C200 (IKA-Werke GmbH & Co. KG, Staufen Breisgau, Germany)) by placing 1.0 g of each biomass sample inside a steel vessel containing 500 µL of deionised water. Then, the vessel was filled with oxygen at 30 bar pressure before it was placed inside the equipment in which the sample was ignited.

### 3.5. Py-GC-MS/FID

Pyrolysis and analysis of the gaseous product from the raw and blended biomass samples were obtained with a micro-pyrolyser (Pyrola2000 (Pyrolab, Lund, Sweden)) connected to a Gas Chromatograph (GC), Mass Spectrometer (MS), and Flame Ionisation Detector (FID), also known as a Py-GC-MS/FID. The biomass samples were blended in different proportions, and close to 500 µg was weighed on an analytical balance (KERN ABT 320-4M) and placed on the platinum filament in the micro-pyrolyser. The platinum filament enables the investigation of primary reactions under isothermal conditions due to its high heating ramp of about 100,000 °C/s and good temperature control. Fast pyrolysis of the raw and blended biomasses was performed at a temperature of 600 °C with a residence time of 5 s. The operating condition was selected in order to obtain the maximum product yield of the released volatile compounds, as reported in previous studies [28,29].

Both the micro-pyrolyser chamber temperature and the transfer tube were set to 150 °C. The low chamber temperature minimises secondary reactions, while it is high enough to keep many of the products in the gas phase. The released pyrolysis vapour was directly transferred to the GC (Trace GC Ultra, Thermo Scientific, Waltham, MA, USA) via a continuous flow of helium gas with a purity of 99.9%. The volatile compounds were identified via the MS (ISQTM, Thermo Scientific) and quantified via an FID (Thermo Scientific). A Zebron™ ZB-5MS (30 m × 0.25 mm × 0.25 µm) capillary column was used with helium carrier gas (1.5 mL min^−1^) and a split ratio of 1:7, while the GC oven programme was set at a temperature ramp of 1 min at 60 °C, followed by a ramp of 8 °C/min to 265 °C, and was then held at 265 °C for 20 min. The MS was operated with an ionisation energy of 70 eV, while the MS transfer line and the FID base temperatures were set to 250 °C. Furthermore, the ion-source temperature was kept at 200 °C, and the MS scan was obtained from 25 to 250 m/z. More than 50 chromatography peaks were extracted by the GC-MS Xcalibur software (Version 2.1.0–2.3.0) and identified by the NIST library (NIST MS Search 2.0) and based on previous research [28,30]. A minimum of three replicates were performed to ensure reproducibility and to minimise errors. Quantification of the released volatile compounds was based on their response in count/µg sample for each of the evolved volatile compounds, as described in a previous study [30]. Moreover, the data presented in this study are based on the released volatile compounds that did not condense in the glass cell of the pyrola. This set-up, thus, shows the potential to reduce secondary reactions due to its high heating ramp and precise temperature control compared to non-isothermal reactors mostly used to investigate the co-pyrolysis of different biomasses.

## 4. Conclusions

The co-pyrolysis of palm kernel shell and sawdust from two different woody biomasses was investigated to understand their influence on the primary-product yield and synergistic interactions.

1. The binary blends show that the co-pyrolysis of PKS with MAH or IRO in equal proportions (PKS:MAH-1:1 and PKS:IRO-1:1) decreased the relative yield of phenolic compounds by 19% compared to the pyrolysis of each material individually;
2. The saccharides, mainly levoglucosan, were inhibited to a large extent, while HAA was promoted by 43% for the PKS:IRO-1:1 pyrolysis blend;
3. The relative yields of 2,6-dimethoxyphenol and furfural were also promoted by 21 and 37%, respectively, for the pyrolysis of the MAH:IRO-1:1 blend;
4. No major difference in the relative yield was observed across the different classes of compounds when the woody biomasses were co-pyrolysed together, which is due to their similar chemical structures;
5. The ternary blends showed that the pyrolysis of PKS, MAH, and IRO in equal proportions (PKS:MAH:IRO_1:1:1) led to an increase in the relative yield of the saccharides to a large extent, while an increase in the proportion of the woody biomass in the pyrolysis blend (PKS:MAH:IRO-1:2:2) led to a strong inhibition in the relative yield of the saccharides;
6. Analysis of the individual volatile compounds formed shows that the pyrolysis of PKS:MAH:IRO-1:2:2 resulted in a decreased yield of phenols by 25%, while the relative yields of HAA and levoglucosan were promoted by 34 and 24%, respectively, for PKS:MAH:IRO_1:1:1.

## Figures and Tables

**Table 1:** Proximate analysis and characterisation of individual biomass feedstocks.

Feedstock	PKS (wt.%)	MAH (wt.%)	IRO (wt.%)
Proximate analysis			
Moisture content	2.2 ± 0.1	3.9 ± 0.1	3.8 ± 0.2
Ash content	0.9 ± 0.1	2.6 ± 0.4	4.8 ± 0.1
Volatile matter	76.7 ± 1.0	82.5 ± 0.6	78.6 ± 0.1
Fixed carbon (by difference)	20.3	11.1	12.9
Calorific value (HHV, MJ/kg)	20.7 ± 0.2	18.9 ± 0.3	19.5 ± 0.2
Component analysis			
Cellulose	8.4 ± 1.3	27.5 ± 0.6	25.0 ± 0.3
Hemicellulose (by difference)	33.5	38.0	31.8
Lignin	57.2 ± 0.7	31.9 ± 1.6	38.4 ± 0.9

## Data Availability

Data can be obtained on request from the corresponding author.

## Supplementary Materials

The following supporting information can be downloaded at https://www.mdpi.com/article/10.3390/molecules28196809/s1, Tables S1–S3: Product distribution of fast pyrolysis of individual and co-pyrolysed biomass blends at 600 °C and 5 s, comparison of the experimental and predicted results of the two-biomass pyrolysis blend, and comparison of the experimental and predicted results of the three-biomass pyrolysis blend.
