# Supplementary material for: Primary Products from Fast Co-Pyrolysis of Palm Kernel Shell and Sawdust
Source: Molecules. 2023 Sep 26;28(19):6809. doi: 10.3390/molecules28196809 (PMC10574147; doi:10.3390/molecules28196809)
Supplement: Supplementary file 1 [file molecules-28-06809-s001.zip › molecules-2596081-supplementary.pdf]

[Supplementary Material]

Primary Products from Fast Co-Pyrolysis of Palm Kernel Shell and Sawdust

David O. Usino *, Päivi Ylitervo and Tobias Richards

Swedish Centre for Resource Recovery, University of Borås, 501 90 Borås, Sweden; paivi.ylitervo@hb.se (P.Y.); tobias.richards@hb.se (T.R.)

* Correspondence: david.usino@hb.se

Table S1. Product distribution of fast pyrolysis of individual and co-pyrolysed biomass blends at 600°C and 5 s.

	**Chemical Compounds**	**Individual biomass**			**Two biomass blend**			**Three biomass blend**		
***RT**	**Phenols**	**PKS**	**MAH**	**IRO**	**PKS:MAH-1:1**	**PKS:IRO-1:1**	**MAH:IRO-1:1**	**PKS:MAH:IRO -1:1:1**	**PKS:MAH:IRO -1:2:2**	**PKS:MAH:IRO -2:1:1**
5.83	**Phenol**	**2.59**	**0.32**	**0.12**	**2.46**	**2.67**	**0.12**	**1.17**	**1.21**	**2.85**
5.94	4-Ethylphenol	0	0	0.21		0	0.3	0.18	0.25	0
7.13	2-Methylphenol	0.17	0.18	0.07	0.26	0.28	0.2	0.22	0.18	0.37
7.54	4-Methylphenol	0.22	0.12	0.17	0.14	0.19	0.11	0.16	0.15	0.23
7.7	2-Methoxyphenol	0.65	0.46	0.68	0.71	0.9	0.71	0.79	0.67	0.87
8.79	2,5-Dimethylphenol	0.16	0.14	0.34	0.07	0.07	0.08	0.35	0.09	0.08
9.23	4-Ethyl-1,3-benzenediol	0	0.24	0.41	0.16	0.11	0.24	0.33	0.13	0.26
9.52	4-Methyl-2-methoxyphenol	0.68	0.16	0.35	0.65	0.89	0.39	0.67	0.54	0.99
9.97	1,2-Benzenediol	0.87	0.43	0.33	0.52	0.5	0.45	0.34	0.47	0.82
10.83	3-Methoxy-1,2-benzenediol	0	0.36	0.27	0	0	0	0.34	0.38	0.39
10.96	4-Ethyl-2-methoxyphenol	0.61	0.36	0.25	0.53	0.53	0.39	0.25	0.18	0.43
11.69	4-Vinyl-2-methoxyphenol	1.15	0.55	1.12	0.98	1.14	0.92	1.33	0.78	1.21
12.27	2,6-Dimethoxyphenol	1.03	0.84	1	1.06	1.03	**1.14**	1.22	0.85	1.33
12.39	3-(2-Propenyl)-2-methoxyphenol	0.09	0	0	0.06	0.06	0.07	0.07	0.07	0.1
12.48	2,4-Dimethoxyphenol	0.22	0.33	0.2	0.15	0.17	0.18	0.1	0.16	0.23
13.12	4-(1-propenyl)- 2-methoxyphenol	0.15	0.07	0.1	0.11	0.12	0.12	0.1	0.09	0.15
13.21	Vanillin	0.31	0.18	0.23	0.19	0.24	0.21	0.27	0.18	0.29
13.77	4-Methoxy-3-(methoxymethyl)phenol	0.68	0.33	0.45	0.78	0.89	0.59	0.64	0.4	1.17
13.84	4-(1-propenyl)- 2-methoxyphenol	0.63	0.37	0.58	0.46	0.54	0.46	0.59	0.33	0.47
14.07	4-Propyl-2-methoxyphenol	0.15	0.1	0.17	0.1	0.09	0.16	0.13	0.12	0.11
14.53	1-(4-Hydroxy-3-methoxyphenyl)-ethanone	0.19	0.17	0.14	0.2	0.23	0.12	0.18	0.19	0.18
14.91	5-tert-Butylpyrogallol	0.18	0.07	0.08	0.08	0.08	0.1	0.13	0.14	0.11
15.13	4-Hydroxy-3-methoxyphenylpropan-2-one	0.23	0.2	0.19	0.19	0.17	0.06	0.2	0.2	0.19
15.59	3-tert-Butyl-4-hydroxyanisole	0.51	0.49	0.99	0.68	0.61	0.74	1.16	0.63	0.95

15.79	4-(3-hydroxy-1-propenyl)-2-methoxyphenol	0.36	0.53	0.26	0.14	0.22	0.15	0.39	0.2	0.24
15.87	3-Hydroxy-4-methoxybenzaldehyde	0	0	0				0.4	0.29	0.32
16.07	4-(2-Propenyl)-2,6-dimethoxyphenol	0.6	0.47	0.31	0.34	0.52	0.26	0.33	0.31	0.44
16.03	4-(2-Propenyl)-2,6-dimethoxyphenol	0	0	0	0.12	0.33	0.1	0.22	0.12	0.17
16.77	4-(2-Propenyl)-2,6-dimethoxyphenol	0.23	0.12	0.12	0.1	0.12	0.1	0	0	0
17.07	4-Hydroxy-3,5-dimethoxybenzaldehyde	0	0.24	0.19	0.09	0.11	0.23	0.37	0.12	0.15
17.57	4-(2-Propenyl)-2,6-dimethoxyphenol	0.68	0.42	0.62	0.63	0.57	0.56	0.7	0.46	0.66
18.05	2,3,4-Trimethoxybenzaldehyde	0	0.09	0.12	0.08	0.08	0.15	0.15	0.08	0.1
18.13	4-Hydroxy-2-methoxycinnamaldehyde	0	0	0.36		0	0.1	0.25	0	0
18.45	4-Hydroxy-3-methoxybenzeneacetic acid	0.34	0.15	0	0.11	0.1	0.11	0.13	0.08	0.16
	**Total Phenols**	**13.69**	**8.49**	**10.44**	**12.16**	**13.59**	**9.63**	**13.82**	**10.04**	**16.03**
	**Ketones**									
2.35	**1-Hydroxy-2-propanone**	**3.44**	**5.2**	**5.91**	**5.7**	**4.93**	**5.31**	**4.36**	**4.48**	**4.68**
2.54	2-Pentanone	0.83	0.88	0.85	0.91	0.94	1.04	0.88	0.84	0.86
3.21	2,3-Pentanedione	1.22	2.14	2.01	1.37	1.54	2.33	2.31	1.51	1.77
4.21	2,6-Dimethyl-1,5-heptadiene	0	0	0.14				0.34	0.42	0.31
4.4	2-Cyclopentene-1,4-dione	0	0.44	0.64	0	0.6	0.59	0.31	0.31	0.34
4.71	2-Methyl-2-cyclopentenone	0.95	0.32	0.24	0	0	0	0.26	0.25	0
5.1	2-Hydroxycyclopent-2-en-1-one	0	0.84	0.92	1.45	1.46	0.99	1.14	1.37	1.33
5.54	3-Methylcyclopentanediol	0	0	0				0.2	0.36	0.5
6.4	4-Hydroxy-5,6-dihydro-2H-pyran-2-one	3.24	1.03	0.93	2.32	2.22	1.35	0.97	1.4	2.66
6.36	3,4-Dihydro-2-methoxy-2H-pyran	0	0	0				1.13	0.67	0.66
6.79	2-Hydroxy-3-methyl-2-cyclopentenone	0.25	0.36	0.54	0.26	0.62	0.51			
8.34	3-Ethyl-2-hydroxy-2-cyclopenten-1-one	0	0	0.43	0.15	0.1	0.32	0.32	0.14	0.2
9.89	2-Methyl-3,5-dihydroxy-4H-pyran-4-one	0	0.47	0.52	0	0	0	0.11		
	**Total Ketones**	**9.93**	**11.68**	**13.12**	**12.17**	**12.42**	**12.44**	**12.33**	**11.76**	**13.3**
	**Aldehydes**									
1.81	Acetaldehyde	16.72	17.94	18.28	11.68	13.65	12.69	12.28	12.46	12.19

2.09	**Hydroxyacetaldehyde**	**33.47**	**42.32**	**42.15**	**33.13**	**27.89**	**47.7**	**39.93**	**34.07**	**24.43**
	**Total Aldehydes**	**50.19**	**60.26**	**60.43**	**44.82**	**41.54**	**60.39**	**52.2**	**46.52**	**36.62**
	**Acids**									
2.22	**Acetic acid**	**16.02**	**11.93**	**9.39**	**23.84**	**24.48**	**11.52**	**12.59**	**24.58**	**25.14**
3.04	2-Methylpropanoic acid	1.99	2.2	2.27	2.22	2.51	2.06	2.21	2.24	2.27
15.94	4-Acetoxybenzoic acid	1.18	1.32	1.1	0.3	0.43	0.21		0.22	0.41
16.24	4-Hydroxybenzoic acid	0.55	0	0	0	0		0.13	0.89	0.79
20.88	Oleic Acid	1.65	0.09	0.07	0.65	0.74	0.08	0.86	0.29	0.96
22.92	n-Hexadecanoic acid	0.65	0.05	0.06	0.36	0.31	0.07	0.41	0.19	0.42
23.33	Dodecanoic acid	0.48	0.31	0.38	0.12	0.12	0.02	0.14	0.06	0.15
	**Total Acid**	**22.52**	**15.91**	**13.27**	**27.49**	**28.58**	**13.97**	**16.33**	**28.47**	**30.15**
	**Sachharides**									
8.31	Levoglucosenone	0	0	0.23		0	0.02	0.18	0.01	0.01
16.34	**Levoglucosan**	**0.8**	**0.84**	**0.13**	**0.53**	**0.72**	**0.66**	**1.43**	**0.32**	**0.8**
	**Total Sachharides**	**0.8**	**0.84**	**0.36**	**0.53**	**0.72**	**0.68**	**1.61**	**0.33**	**0.81**
	**Furans**									
3.71	**Furfural**	**1.54**	**0.98**	**1.43**	**1.88**	**2.11**	**1.82**	**1.53**	**1.84**	**1.88**
4.05	2,5-Dimethyl-2,3-dihydrofuran	0.72	1	0.23	0.68	0.55	0.66	0.77	0.54	0.51
4.97	2(3H)-furanone	0	0.24	0	0		0	0.41	0.18	0.25
5.66	3-Butyldihydro-2(3H)-furanone	0.61	0.6	0.46	0.29	0.49	0.41	0.58	0.16	0.34
10.1	2,3-Dihydrobenzofuran	0	0	0.26		0	0	0.43	0	0
	**Total Furans**	**2.87**	**2.82**	**2.38**	**2.85**	**3.16**	**2.89**	**3.72**	**2.72**	**2.98**

Highlighted compounds refer to main chemical compounds formed in each group of compounds.

*RT. - Retention time (mins).

Product distribution is given in normalised response (count/µg sample).

Table S2. Comparison of the experimental and predicted results of the two-biomass pyrolysis blend

PKS_MAH_1:1	Response (count/µg sample)		STDEV	STDEV
**Group of chemical compounds**	**Exp.**	**Pred.**	**Exp.**	**Pred.**
**Phenols**	8794	6483	362	312
**Ketones**	9200	5946	1391	399
**Aldehydes**	33895	19157	8385	3703
**Acids**	20787	11097	1921	1296
**Sachharides**	425	470	24	126
**Furans**	2152	1650	194	262
	**75253**	**44803**		

PKS_IRO_1:1	Response (count/µg sample)		STDEV	STDEV
**Group of chemical compounds**	**Exp.**	**Pred.**	**Exp.**	**Pred.**
**Phenols**	10000	5974	481	303
**Ketones**	9139	5062	562	351
**Aldehydes**	30574	13714	2722	2278
**Acids**	21036	9299	4041	797
**Sachharides**	532	316	303	88
**Furans**	2325	1334	346	243
	**73606**	**35699**		

MAH_IRO_1:1	Response (count/µg sample)		STDEV	STDEV
**Group of chemical compounds**	**Exp.**	**Pred.**	**Exp.**	**Pred.**
**Phenols**	6345	3318	163	158
**Ketones**	8195	4377	613	352
**Aldehydes**	39790	21711	5910	3452
**Acids**	9205	5370	459	1107
**Sachharides**	435	237	262	92
**Furans**	1907	956	230	116
	**65876**	**35970**		

Table S3. Comparison of the experimental and predicted results of the three-biomass pyrolysis blend

PKS_MAH_IRO_1:1:1	Response (count/µg sample)		STDEV	STDEV
**Group of chemical compounds**	**Exp.**	**Pred.**	**Exp.**	**Pred.**
**Phenols**	5412	5870	184	308
**Ketones**	4996	5103	307	367
**Aldehydes**	20456	18194	2320	3205
**Acids**	6399	8028	178	1075
**Sachharides**	628	321	111	104
**Furans**	1458	1314	115	217
	**39350**	**38829**		

PKS_MAH_IRO_1:2:2	Response (count/µg sample)		STDEV	STDEV
**Group of chemical compounds**	**Exp.**	**Pred.**	**Exp.**	**Pred.**
**Phenols**	8125	5028	505	346
**Ketones**	9518	4797	531	464
**Aldehydes**	37647	19601	8026	4268
**Acids**	23035	6786	5076	1402
**Sachharides**	257	297	46	128
**Furans**	2330	1217	197	239
	**80912**	**37725**		

PKS_MAH_IRO_2:1:1	Response (count/µg sample)		STDEV	STDEV
**Group of chemical compounds**	**Exp.**	**Pred.**	**Exp.**	**Pred.**
**Phenols**	12382	6923	403	407
**Ketones**	10276	5484	801	433
**Aldehydes**	28287	16435	2365	3550
**Acids**	23285	9580	1816	1226
**Sachharides**	621	386	249	126
**Furans**	2385	1521	243	293
	**77236**	**40329**		
